# Supplementary material for: Audiovisual cues must be predictable and win-paired to drive risky choice
Source: eLife. 2026 Apr 13;14:RP105951. doi: 10.7554/eLife.105951 (PMC13075936; doi:10.7554/eLife.105951)
Supplement: Supplementary file 1. [file elife-105951-supp1.docx]

| **Table A: P1-P4 choice comparisons**  **Tukey HSD** | | | | |
| --- | --- | --- | --- | --- |
| **Task comparison** | | | **Mean difference** | **Significance** |
| **P1** | Uncued | Standard | **0.17** | 0.007 |
|  |  | Reverse | *0.14* | 0.06 |
|  |  | Outcome | **0.18** | 0.006 |
|  |  | Random | 0.09 | 0.45 |
|  |  | Loss | 0.08 | 0.62 |
|  | Standard | Reverse | -0.03 | 1.00 |
|  |  | Outcome | 0.01 | 1.00 |
|  |  | Random | -0.08 | 0.62 |
|  |  | Loss | -0.09 | 0.45 |
|  | Reverse | Outcome | 0.04 | 0.98 |
|  |  | Random | -0.05 | 0.93 |
|  |  | Loss | -0.06 | 0.83 |
|  | Outcome | Random | -0.09 | 0.54 |
|  |  | Loss | -0.10 | 0.38 |
|  | Random | Loss | -0.01 | 1.00 |
| **P2** | Uncued | Standard | 0.20 | 0.52 |
|  |  | Reverse | 0.06 | 1.00 |
|  |  | Outcome | 0.21 | 0.48 |
|  |  | Random | -0.07 | 0.99 |
|  |  | Loss | -0.20 | 0.52 |
|  | Standard | Reverse | -0.14 | 0.85 |
|  |  | Outcome | 0.01 | 1.00 |
|  |  | Random | -0.27 | 0.20 |
|  |  | Loss | **-0.40** | 0.01 |
|  | Reverse | Outcome | 0.15 | 0.81 |
|  |  | Random | -0.13 | 0.89 |
|  |  | Loss | -0.26 | 0.28 |
|  | Outcome | Random | -0.28 | 0.19 |
|  |  | Loss | **-0.41** | 0.01 |
|  | Random | Loss | -0.13 | 0.90 |
| **P3** | Uncued | Standard | -0.16 | 0.47 |
|  |  | Reverse | -0.20 | 0.26 |
|  |  | Outcome | **-0.35** | 0.004 |
|  |  | Random | -0.04 | 0.99 |
|  |  | Loss | 0.08 | 0.95 |
|  | Standard | Reverse | -0.04 | 0.99 |
|  |  | Outcome | -0.19 | 0.35 |
|  |  | Random | 0.13 | 0.76 |
|  |  | Loss | 0.25 | 0.10 |
|  | Reverse | Outcome | -0.15 | 0.65 |
|  |  | Random | 0.17 | 0.53 |
|  |  | Loss | **0.29** | 0.04 |
|  | Outcome | Random | **0.32** | 0.02 |
|  |  | Loss | **0.44** | 0.0002 |
|  | Random | Loss | 0.12 | 0.82 |
| **P4** | Uncued | Standard | -0.14 | 0.37 |
|  |  | Reverse | 0.06 | 0.98 |
|  |  | Outcome | 0.02 | 1.00 |
|  |  | Random | 0.11 | 0.72 |
|  |  | Loss | 0.14 | 0.45 |
|  | Standard | Reverse | 0.20 | 0.09 |
|  |  | Outcome | 0.16 | 0.28 |
|  |  | Random | **0.25** | 0.01 |
|  |  | Loss | **0.28** | 0.004 |
|  | Reverse | Outcome | -0.04 | 0.99 |
|  |  | Random | 0.05 | 0.99 |
|  |  | Loss | 0.08 | 0.90 |
|  | Outcome | Random | 0.09 | 0.86 |
|  |  | Loss | 0.12 | 0.63 |
|  | Random | Loss | 0.03 | 0.99 |

Table A: Comparisons of P1-P4 between task variants using Tukey’s honest significant differences (HSD) test. Bolded values indicate a significant difference, italicized values indicate a trending difference.

| **Table B: Premature responding comparisons**  **Tukey HSD** | | | | |
| --- | --- | --- | --- | --- |
| **Task comparison** | | | **Mean difference** | **Significance** |
|  | Uncued | Standard | -0.02 | 1.00 |
|  |  | Reverse | 0.08 | 0.16 |
|  |  | Outcome | -0.03 | 0.96 |
|  |  | Random | **0.10** | 0.02 |
|  |  | Loss | **0.24** | <0.0001 |
|  | Standard | Reverse | **0.10** | 0.05 |
|  |  | Outcome | -0.01 | 1.00 |
|  |  | Random | **0.12** | 0.005 |
|  |  | Loss | **0.25** | <0.0001 |
|  | Reverse | Outcome | **-0.11** | 0.02 |
|  |  | Random | 0.02 | 0.98 |
|  |  | Loss | **0.16** | <0.0001 |
|  | Outcome | Random | **0.13** | 0.002 |
|  |  | Loss | **0.27** | <0.0001 |
|  | Random | Loss | **0.13** | 0.001 |

Table B: Comparisons of premature responding between task variants using Tukey’s HSD test. Bolded values indicate a significant difference.

| **Table C: Collect latency comparisons**  **Tukey HSD** | | | | |
| --- | --- | --- | --- | --- |
| **Task comparison** | | | **Mean difference** | **Significance** |
|  | Uncued | Standard | 0.13 | 0.93 |
|  |  | Reverse | 0.05 | 1.00 |
|  |  | Outcome | -0.09 | 0.99 |
|  |  | Random | **-0.72** | <0.0001 |
|  |  | Loss | -0.02 | 1.00 |
|  | Standard | Reverse | -0.08 | 0.99 |
|  |  | Outcome | -0.21 | 0.61 |
|  |  | Random | **-0.84** | <0.0001 |
|  |  | Loss | -0.14 | 0.90 |
|  | Reverse | Outcome | -0.13 | 0.93 |
|  |  | Random | **-0.76** | <0.0001 |
|  |  | Loss | -0.06 | 1.00 |
|  | Outcome | Random | **-0.63** | 0.0002 |
|  |  | Loss | 0.07 | 1.00 |
|  | Random | Loss | **0.70** | <0.0001 |

Table C: Comparisons of collect latency between task variants using Tukey’s HSD test. Bolded values indicate a significant difference.

| **Table D: Nonlinear model simulated decision score comparisons**  **Tukey HSD** | | | | |
| --- | --- | --- | --- | --- |
| **Task comparison** | | | **Mean difference** | **Significance** |
|  | Uncued | Standard | **43.58** | 0.002 |
|  |  | Reverse | 29.40 | 0.12 |
|  |  | Outcome | **36.04** | 0.03 |
|  |  | Random | 5.22 | 0.99 |
|  |  | Loss | 4.72 | 0.99 |
|  | Standard | Reverse | -14.18 | 0.77 |
|  |  | Outcome | -7.55 | 0.98 |
|  |  | Random | **-38.37** | 0.006 |
|  |  | Loss | **-38.86** | 0.005 |
|  | Reverse | Outcome | 6.63 | 0.99 |
|  |  | Random | -24.19 | 0.25 |
|  |  | Loss | -24.68 | 0.23 |
|  | Outcome | Random | *-30.82* | 0.08 |
|  |  | Loss | *-31.32* | 0.07 |
|  | Random | Loss | -0.50 | 0.99 |

Table D: Comparisons of decision scores simulated from nonlinear model subject-level parameter estimates using Tukey’s HSD test. Bolded values indicate a significant difference, italicized values indicate a trending difference.

| **Table E: Scaled + offset model simulated decision score comparisons**  **Tukey HSD** |
| --- |

| **Task comparison** | | | **Mean difference** | **Significance** |
| --- | --- | --- | --- | --- |
|  | Uncued | Standard | **36.63** | 0.01 |
|  |  | Reverse | 26.86 | 0.18 |
|  |  | Outcome | *32.53* | 0.07 |
|  |  | Random | 5.52 | 0.99 |
|  |  | Loss | 6.02 | 0.99 |
|  | Standard | Reverse | -9.77 | 0.94 |
|  |  | Outcome | -4.10 | 0.99 |
|  |  | Random | **-31.10** | 0.04 |
|  |  | Loss | **-30.61** | 0.05 |
|  | Reverse | Outcome | 5.67 | 0.99 |
|  |  | Random | -21.34 | 0.37 |
|  |  | Loss | -20.84 | 0.40 |
|  | Outcome | Random | -27.00 | 0.16 |
|  |  | Loss | -26.51 | 0.18 |
|  | Random | Loss | 0.50 | 0.99 |

| Table E: Comparisons of decision scores simulated from scaled + offset model subject-level parameter estimates using Tukey’s HSD test. Bolded values indicate a significant difference.   \| **Table F: Nonlinear model simulated P1-P4 choice comparisons Tukey HSD** \| \| \| \| \| \| --- \| --- \| --- \| --- \| --- \| \| **Task comparison** \| \| \| **Mean difference** \| **Significance** \| \| **P1** \| Uncued \| Standard \| **0.30** \| <0.0001 \| \| Reverse \| 0.17 \| 0.09 \| \| Outcome \| **0.24** \| 0.01 \| \| Random \| 0.15 \| 0.18 \| \| Loss \| 0.03 \| 0.99 \| \| Standard \| Reverse \| -0.13 \| 0.26 \| \| Outcome \| -0.07 \| 0.89 \| \| Random \| -0.15 \| 0.13 \| \| Loss \| **-0.27** \| <0.0001 \| \| Reverse \| Outcome \| 0.06 \| 0.92 \| \| Random \| -0.02 \| 1.00 \| \| Loss \| -0.14 \| 0.24 \| \| Outcome \| Random \| -0.08 \| 0.78 \| \| Loss \| **-0.20** \| 0.02 \| \| Random \| Loss \| -0.12 \| 0.40 \| \| **P2** \| Uncued \| Standard \| -0.02 \| 1.00 \| \| Reverse \| 0.02 \| 1.00 \| \| Outcome \| -0.01 \| 1.00 \| \| Random \| -0.10 \| 0.58 \| \| Loss \| -0.01 \| 1.00 \| \| Standard \| Reverse \| 0.04 \| 0.98 \| \| Outcome \| 0.01 \| 1.00 \| \| Random \| -0.08 \| 0.73 \| \| Loss \| 0.01 \| 1.00 \| \| Reverse \| Outcome \| -0.03 \| 1.00 \| \| Random \| -0.12 \| 0.33 \| \| Loss \| -0.03 \| 1.00 \| \| Outcome \| Random \| -0.10 \| 0.65 \| \| Loss \| 0.00 \| 1.00 \| \| Random \| Loss \| 0.10 \| 0.61 \| \| **P3** \| Uncued \| Standard \| **-0.17** \| 0.001 \| \| Reverse \| -0.08 \| 0.39 \| \| Outcome \| **-0.15** \| 0.01 \| \| Random \| -0.02 \| 1.00 \| \| Loss \| -0.03 \| 0.96 \| \| Standard \| Reverse \| 0.09 \| 0.17 \| \| Outcome \| 0.02 \| 1.00 \| \| Random \| **0.15** \| 0.001 \| \| Loss \| **0.13** \| 0.01 \| \| Reverse \| Outcome \| -0.07 \| 0.46 \| \| Random \| 0.06 \| 0.60 \| \| Loss \| 0.04 \| 0.86 \| \| Outcome \| Random \| **0.14** \| 0.01 \| \| Loss \| **0.12** \| 0.04 \| \| Random \| Loss \| -0.02 \| 1.00 \| \| **P4** \| Uncued \| Standard \| **-0.19** \| 0.004 \| \| Reverse \| -0.12 \| 0.19 \| \| Outcome \| **-0.16** \| 0.04 \| \| Random \| -0.02 \| 1.00 \| \| Loss \| -0.03 \| 0.99 \| \| Standard \| Reverse \| 0.07 \| 0.73 \| \| Outcome \| 0.03 \| 0.99 \| \| Random \| **0.17** \| 0.01 \| \| Loss \| **0.16** \| 0.01 \| \| Reverse \| Outcome \| -0.04 \| 0.97 \| \| Random \| 0.10 \| 0.39 \| \| Loss \| 0.09 \| 0.43 \| \| Outcome \| Random \| 0.14 \| 0.09 \| \| Loss \| 0.13 \| 0.10 \| \| Random \| Loss \| 0.00 \| 1.00 \|   Table F: Comparisons of P1-P4 simulated from nonlinear model subject-level parameter estimates using Tukey’s HSD test. Bolded values indicate a significant difference. |
| --- | --- | --- | --- | --- | --- | --- | --- | --- | --- | --- | --- | --- | --- | --- | --- | --- | --- | --- | --- | --- | --- | --- | --- | --- | --- | --- | --- | --- | --- | --- | --- | --- | --- | --- | --- | --- | --- | --- | --- | --- | --- | --- | --- | --- | --- | --- | --- | --- | --- | --- | --- | --- | --- | --- | --- | --- | --- | --- | --- | --- | --- | --- | --- | --- | --- | --- | --- | --- | --- | --- | --- | --- | --- | --- | --- | --- | --- | --- | --- | --- | --- | --- | --- | --- | --- | --- | --- | --- | --- | --- | --- | --- | --- | --- | --- | --- | --- | --- | --- | --- | --- | --- | --- | --- | --- | --- | --- | --- | --- | --- | --- | --- | --- | --- | --- | --- | --- | --- | --- | --- | --- | --- | --- | --- | --- | --- | --- | --- | --- | --- | --- | --- | --- | --- | --- | --- | --- | --- | --- | --- | --- | --- | --- | --- | --- | --- | --- | --- | --- | --- | --- | --- | --- | --- | --- | --- | --- | --- | --- | --- | --- | --- | --- | --- | --- | --- | --- | --- | --- | --- | --- | --- | --- | --- | --- | --- | --- | --- | --- | --- | --- | --- | --- | --- | --- | --- | --- | --- | --- | --- | --- | --- | --- | --- | --- | --- | --- | --- | --- | --- | --- | --- | --- | --- | --- | --- | --- | --- | --- | --- | --- | --- | --- | --- |
|  |
|  |

| **Table G: Scaled + offset model simulated P1-P4 choice comparisons**  **Tukey HSD** | | | | |
| --- | --- | --- | --- | --- |
| **Task comparison** | | | **Mean difference** | **Significance** |
| **P1** | Uncued | Standard | **0.25** | 0.001 |
|  |  | Reverse | **0.18** | 0.04 |
|  |  | Outcome | **0.20** | 0.02 |
|  |  | Random | 0.15 | 0.18 |
|  |  | Loss | 0.02 | 1.00 |
|  | Standard | Reverse | -0.07 | 0.85 |
|  |  | Outcome | -0.05 | 0.96 |
|  |  | Random | -0.10 | 0.47 |
|  |  | Loss | **-0.23** | 0.002 |
|  | Reverse | Outcome | 0.02 | 1.00 |
|  |  | Random | -0.04 | 0.99 |
|  |  | Loss | *-0.16* | 0.08 |
|  | Outcome | Random | -0.06 | 0.95 |
|  |  | Loss | **-0.18** | 0.04 |
|  | Random | Loss | -0.13 | 0.30 |
| **P2** | Uncued | Standard | -0.01 | 1.00 |
|  |  | Reverse | 0.00 | 1.00 |
|  |  | Outcome | 0.00 | 1.00 |
|  |  | Random | -0.09 | 0.73 |
|  |  | Loss | 0.01 | 1.00 |
|  | Standard | Reverse | 0.01 | 1.00 |
|  |  | Outcome | 0.02 | 1.00 |
|  |  | Random | -0.08 | 0.78 |
|  |  | Loss | 0.03 | 1.00 |
|  | Reverse | Outcome | 0.01 | 1.00 |
|  |  | Random | -0.09 | 0.71 |
|  |  | Loss | 0.02 | 1.00 |
|  | Outcome | Random | -0.10 | 0.68 |
|  |  | Loss | 0.01 | 1.00 |
|  | Random | Loss | 0.11 | 0.56 |
| **P3** | Uncued | Standard | **-0.14** | 0.02 |
|  |  | Reverse | -0.11 | 0.15 |
|  |  | Outcome | **-0.15** | 0.02 |
|  |  | Random | -0.01 | 1.00 |
|  |  | Loss | -0.05 | 0.89 |
|  | Standard | Reverse | 0.03 | 0.98 |
|  |  | Outcome | -0.01 | 1.00 |
|  |  | Random | **0.12** | 0.04 |
|  |  | Loss | 0.09 | 0.29 |
|  | Reverse | Outcome | -0.04 | 0.97 |
|  |  | Random | 0.10 | 0.25 |
|  |  | Loss | 0.06 | 0.73 |
|  | Outcome | Random | **0.13** | 0.04 |
|  |  | Loss | 0.10 | 0.26 |
|  | Random | Loss | -0.03 | 0.97 |
| **P4** | Uncued | Standard | **-0.17** | 0.009 |
|  |  | Reverse | -0.09 | 0.46 |
|  |  | Outcome | **-0.15** | 0.05 |
|  |  | Random | -0.03 | 0.99 |
|  |  | Loss | -0.04 | 0.97 |
|  | Standard | Reverse | 0.08 | 0.56 |
|  |  | Outcome | 0.02 | 1.00 |
|  |  | Random | **0.14** | 0.04 |
|  |  | Loss | *0.13* | 0.06 |
|  | Reverse | Outcome | -0.06 | 0.87 |
|  |  | Random | 0.06 | 0.81 |
|  |  | Loss | 0.05 | 0.89 |
|  | Outcome | Random | 0.12 | 0.18 |
|  |  | Loss | 0.11 | 0.25 |
|  | Random | Loss | -0.01 | 1.00 |

Table G: Comparisons of P1-P4 simulated from scaled + offset model subject-level parameter estimates using Tukey’s HSD test. Bolded values indicate a significant difference, italicized values indicate a trending difference.
